# Edible Coating Formulated by Optimization from *Aloe vera*, Starch, and Arabic Gum Improved the Conservation of Banana (*Musa acuminata*) Fruits

**DOI:** 10.1155/2023/3746425

**Published:** 2023-09-08

**Authors:** Alain Ngotio Tchinda, Mariette Anoumaa, Franck Laurins Tchouala Tazo, Eugène Tafre Phounzong, Odelonne Justine Kenfack, Martin Lekeufack, Roger Braogue Doumdi, Jean Aghofack Nguemezi, Théophile Fonkou

**Affiliations:** ^1^Department of Plant Biology, Faculty of Sciences, University of Dschang, P.O. Box 67, Dschang, Cameroon; ^2^Department of Biochemistry, Faculty of Sciences, University of Dschang, P.O. Box 67, Dschang, Cameroon

## Abstract

Banana is a very perishable climacteric fruit with consequence of large postharvest losses. The objective of the present study was to improve the postharvest shelf life of bananas. Fruits from the Melong locality were treated with coating solutions formulated with a mixture of *Aloe vera*, starch, and Arabic gum at different concentrations. These concentrations were obtained using the response surface methodology in order to establish the relationship between independent variables (ripening parameters) and dependent variable (*Aloe vera*, starch and Arabic gum), which led to the generation of experimental design as well as the prediction of result model. The effects of the coating solutions were evaluated on firmness, percentage of ripening, weight loss, chlorophyll *a*, chlorophyll *b*, and total soluble solid contents. The results showed that the combination of *Aloe vera*, starch, and Arabic gum extended the shelf life of banana by slowing down the chlorophyll degradation, the loss of firmness, the weight loss, and the synthesis of total soluble solids. The coefficients of determination (*R*^2^) of the responses were all above 80% indicating that the experimental data fit well with the predicted responses. The interactions that influenced most of the responses were those between *Aloe vera*-starch and *Aloe vera*-Arabic gum. The optimum concentrations obtained for the mixture of the final solution were 286.799 ml.l^−1^ (*v*/*v*), 102.589 g.l^−1^ (*m*/*v*), and 1.0888% (*m*/*v*) for *Aloe vera*, starch, and Arabic gum, respectively.

## 1. Introduction

Banana is the fourth most important food crop in the world after rice, wheat, and maize [1]. It ranks first in fruit production, with over 106 million tons produced annually worldwide [2]. Banana is imported mainly by the European Union and the United States which record 32% and 26%, respectively, of the total estimated export of 19.4 million tons in 2019 [3]. The main banana-exporting continents in the world are Asia, Latin America, and Africa. In Africa, Cameroon is the second-largest exporter after Ivory Coast with 167,000 tons in 2019 [3]. In Cameroon, banana is produced on more than 7,000 hectares with an average production of 40 t.ha^−1^ [4]. Its strong demand in the European market has boosted the country's exports from 280,000 tons in 2009 to 305,000 tons in 2016 [5].

Banana fruit is essential starchy food rich in vitamins of the groups A, B, C, and E and also in minerals (iron, zinc, calcium, and magnesium). In addition, it is an important source of antioxidants such as carotenes, dopamine, phenols, and flavonoids [6].

Banana is a climacteric fruit generally harvested in its mature green state. It is vulnerable to rapid deterioration because it is a living product with ongoing metabolic activities during storage. At a room temperature, the shelf life of the banana is very limited. According to Subramanyam [7, 8], ripening signs appear 6 to 7 days after harvesting. Thus, storage and exports of bananas are very difficult because of postharvest losses due to rapid ripening. To date, several conservation techniques have been developed, but the problems of postharvest fruit losses persist. Indeed, postharvest losses are between 15 and 50% in developing countries, with a minimum estimate of 20% at each stage of marketing [9].

The conservation methods already developed include refrigeration in cold rooms, chemical treatments [10, 11], heat treatments [12, 13], controlled and modified atmospheres, and addition of antioxidants [14]. These methods have limitations such as the high cost for the installation of cold rooms, the sensitivity of fruits to lower temperatures, the harmful effect of certain chemicals on consumer health, or the modification of the fruit organoleptic properties. The preservation of fruits in a fresh state therefore requires the use of natural processes without any real change in storage temperatures. Thus, edible coatings appear as an adequate alternative in the preservation of fruits in the fresh state. This preservative property lies in the availability of bioactive compounds in plant extracts.

*Aloe vera* extracts are rich in flavonoids, phenols, glutathione, and *α*-tocopherol [15]. These secondary compounds with their antioxidant properties [16–18] are effective in slowing down the ripening process [18]. Starch is a molecule made-up of polysaccharides [19], with plasticizer properties that protect the integrity of biological membranes. It is from this membrane protection that the starch-based coating applied to plums slowed down their rapid degradation [20]. Arabic gum is the exudate of the stems or branches of *Acacia* trees and is widely used in industrial sectors due to its encapsulation and film-forming properties [21]. Zapata et al. [22] showed that Arabic gum slowed down the ripening process of tomatoes by reducing the respiratory intensity and ethylene production. Simultaneous use of *Aloe vera* gel, starch, and Arabic gum in the formulation of an edible coating solution could for this purpose allow a longer preservation period of fresh bananas.

Therefore, this study is aimed at determining the optimal concentrations of *Aloe vera* gel, starch, and Arabic gum extracts to improve the shelf life of banana using the response surface methodology (RSM).

## 2. Material and Methods

### 2.1. Plant Material

Banana fruit of the Cavendish variety harvested at the mature green stage 92 days after flowering (physiological maturity) in the locality of Melong (Littoral Region of Cameroon) was used in this study. The second hands of the banana bunches were carefully selected, and the fingers were sorted for uniformity of sizes and color. The selected bananas were soaked for 2 minutes in a 0.2% sodium hypochlorite solution and then dried at ambient temperature (24 ± 2°C) to eliminate microbial germs.

### 2.2. Coating Solution Ingredients

Gel from *Aloe vera*, starch, and Arabic gum were used to prepare the coating material. *Aloe vera* gel was extracted from the leaves of at least 3-year-old plants harvested in the Dschang locality. Healthy-looking fresh leaves of *Aloe vera* were harvested and washed with distilled water. After removing the skin using a knife, the remaining from the peeled leave which is made-up of gel was homogenized using a blender. The starch was extracted from fresh cassava (*Manihot esculenta*) tubers using the following method: healthy cassava tubers were peeled, washed, and grated. The paste was mixed with water, and the resulting aqueous solution was filtered using a mesh filter of 1.28 mm-pore diameter. The filtrate obtained was left to settle for 6 hours, then the supernatant was removed and the starch deposited at the bottom of the vessel was dried at 40°C for 2 days [23]. The Arabic gum used was harvested on *Acacia* trees in the locality of Garoua (Cameroon).

### 2.3. Generation of the Experimental Design

The response surface methodology (RSM) was used through the three-factor composite centered design (CCD) to generate the experimental setup. This allowed the evaluation of the effect of three independent variables (*Aloe vera* gel concentration, starch concentration, and Arabic gum concentration) on the responses of the percentage of ripening (PR), firmness, weight Loss (WL), pigment contents, and total soluble solids (TSS) content.

The extreme values (maximum and minimum) of the independent variables used in this design were set based on previous works and preliminary tests (Table 1). In addition, the experimental design generated by the CCD gave 20 treatments among which 6 replicates of the central point are shown on Table 2 [24].

The study of the relationship between the independent and dependent variables as well as the prediction of the optimal concentrations was done following the development of a second-order polynomial function for each response: *Y*_1_ (CHL *a*), *Y*_2_ (CHL *b*), *Y*_3_ (carotenoids), *Y*_4_ (firmness), *Y*_5_ (TSS), *Y*_6_ (RP), and *Y*_7_ (WL). The general formula of the second polynomial equation is given by the following mathematical formula:

(1) $$\begin{array}{c} Y_{i}=a_{0}+a_{1}A+a_{2}B+a_{3}C+a_{11}A^{2}+a_{22}B^{2}+a_{33}C^{2}+a_{12}AB+a_{13}AC+a_{23}BC. \end{array}$$

where *Y*_*i*_ represents the response, *a*_0_ is a constant; *a*_*i*_, *a*_*ii*_, and *a*_*ij*_ are the values of the linear, quadratic, and interaction coefficients, respectively. *A*, *B*, and *C* are the independent variables.

Each of the 21 treatments in Table 2 consisted of 3 replicates in a complete randomized design with 15 bananas in each experimental unit. Bananas of each treatment were placed on the bench at 3 cm distance from each other.

### 2.4. Preparation and Application of the Coating Solution

The starch was introduced into a flat-bottom flask to which 1.5 l of distilled water were added, and the mixture was stirred continuously and brought to a boil. After cooling at room temperature for 15 minutes, 1.5 l of ethanol (96%) were added as well as *Aloe vera* gel and Arabic Gum. The whole mixture was left at room temperature for 15 hours before soaking the fruits. The final coating solution was slightly heavy, allowing to form a thin layer of one micrometer thickness on the banana after coating. All solutions were prepared following the same protocol, using the different concentrations of *Aloe vera* gel, starch, and Arabic gum contained in the experimental design (Table 2). For each treatment T1 to T20, the fruits were soaked for 30 minutes in the coating solution. Untreated fruits were consider at control. Each consisted of 3 replicates in a complete randomized design with 15 bananas in each experimental unit. Bananas of each treatment were placed on the bench at 3 cm distance from each other and stored for 17 days at a room temperature of 24 ± 2°C and a relative humidity of 80 ± 2%.

### 2.5. Evaluation of Effect of Coating Solutions

The effect of different coating solutions was evaluated on the 17th day after treatment (date at which the control fruits started their senescence). The parameters investigated included the ripening percentage (RP), firmness, weight loss (WL), pH, and pigment (chlorophyll *a*, chlorophyll *b*, and carotenoids) contents.

#### 2.5.1. Ripening Percentage, Firmness, and Weight Loss

**Figure 1:**
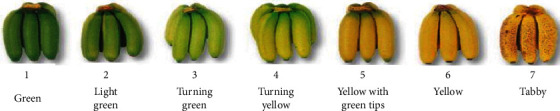
Ripening stages of banana fruits [1].

The ripening percentage (RP) was estimated by counting the ripen fruits at stage 6 (Figure 1). The ripening percentage was calculated using the following formula:

(2) $$\begin{array}{c} \text{RP}=\frac{\text{number of ripen fruits}}{\text{total number of fruits }}\times 100. \end{array}$$

In order to evaluate the firmness of the fruits, the banana skin was removed using a razor blade, and then the cylindrical tip of a GY-2 penetrometer (Sauter GmbH, Germany) was introduced in the pulp to measure the firmness.

Initial fruit mass and the mass of fruit on the day of observation were obtained by weighing bananas on a WH-B05 brand scale and used to calculate the weight loss by the following formula developed by Gharezi et al. [25]

(3) $$\begin{array}{c} \text{WL}=\frac{\text{ initial fruit mass}-\text{mass of fruit on the day of observation}}{\text{ initial fruit mass}}\times 100 \end{array}$$

#### 2.5.2. Pigments and Total Soluble Solid Contents

Pigments concentrations were determined according to the method developed by Lichtenthaler [26]. A 4 g sample of fresh banana peel previously ground with 1 g of sand was introduced into a test tube and mixed which 10 ml of acetone. In addition, the whole tube was wrapped with a light-proof aluminum foil and left to rest in an ice tank for extraction. The absorbances of the extracts were measured at 470 nm, 645 nm, and 662 nm using a Biochrom Libra S22 spectrophotometer. The different pigment contents were calculated by the following formulas.

(4) $$\begin{array}{c} \text{Chlorophyll a}\left(\text{mg}.100\;{\text{ml}}^{-1}\right)=11.24{\text{A}}_{662}-2.04{\text{A}}_{645},\\ \text{Chlorophyll b}\left(\text{mg}.100\;{\text{ml}}^{-1}\right)=20.13{\text{A}}_{645}-4.19{\text{A}}_{662},\\ \text{Carotenoids }\left(\text{mg}.100\;{\text{ml}}^{-1}\right)=\frac{1000{\text{A}}_{470}-1.90\text{Chla}-63.14\text{Chlb}}{214}. \end{array}$$

Total soluble solid content was determined by refractometry following the method described by Dadzie and Orchard [27]. Few drops of previously prepared banana juice were taken and placed on the prism of the ATC-1C refractometer. By pointing the prism towards a light source, the value of TSS content was read.

### 2.6. Optimization and Validation of the Procedure

In order to better visualize the interaction effects of the concentrations of *Aloe vera*, starch, and Arabic gum on the responses, the three-dimensional graphs were generated from the fitted models for each response. Furthermore, referring to the principles of ripening physiology, the optimization of the independent variables was done by a desirability function approach, i.e., at maximum values of chlorophyll *a*, chlorophyll *b*, firmness, and at minimum values of carotenoids, RP, TSS, and WL. For verification and adequacy of the final response (optimal concentrations), new bananas were coated with a coating solution obtained using independent variables at found optimal concentrations.

### 2.7. Statistical Analysis

The modeling, the optimization procedure, the various graphs, and the analysis of all the data collected in this study were done using Minitab software version 2018. The analysis of variance (ANOVA) was performed to compare the effect of different treatments as well as the quality of the effects of the variables of the developed models. Thus, the coefficients of the polynomial equations were calculated from the experimental data. The lack of fit test and analysis of coefficient of determination (*R*^2^) were performed to determine the adequacy of the model [28]. The significance of the linear, quadratic, and interaction effects of the different factors, as well as that of each of these coefficients, was determined by comparing the observed probability (*p* value) to a critical probability (*p* = 0.05). Then, a Tukey's comparison test was performed between the predicted and experimental response values [28].

## 3. Results and Discussion

### 3.1. Model Adjustment

Chlorophyll *a* and chlorophyll *b* contents as well as the firmness values were lower in the control than in all the treated bananas (Table 3). In the same way, the values in total soluble solids, the ripening percentage, and the weight loss of the control were higher than those of the coated bananas. Similar results were reported by Farina et al. [29] on papayas coated with *Aloe vera* gel as well as by Tchouala et al. [30] on tomato coated with a solution made from Arabic gum, starch, and coffee leaf extracts. Bioactive compounds in the solution may have delayed the ripening process of bananas, by slowing down the related metabolic processes (degradation of pigments, decrease in firmness, synthesis of sugars, and weight loss). The highest chlorophyll *a* content (0.427 mg/g) was obtained in bananas treated with 250 ml/l of *Aloe vera*, 150 g/l of starch, and 1% of Arabic gum. The highest values of chlorophyll *b* content (0.189 mg/g), firmness (4.0 N), as well as the lowest values of the ripening percentage (36.66%), and total soluble solid content (11.0°Brix) were found in bananas treated with 290,453 ml/l of *Aloe vera*, 100 g/l of starch, and 1.5% of Arabic gum. Bananas treated with 200 ml/l of *Aloe vera*, 100 g/l of starch, and 1.5% of Arabic gum showed the lowest weight loss (5.921%) (Table 3). These results showed that various concentrations of the ingredients in coating solutions allowed to improve different parameters, whereas the objective is to determine the concentrations that can improve all the parameters. Hence, optimization of these ingredients' concentrations may enable to converge the effects to an appropriate treatment.

### 3.2. Analysis of Variance and Model Validation

The probability values of the linear, interaction, and quadratic effects of the independent variables and the regression coefficients are presented in Table 3. It can be seen that the linear effects of factors A, B, and C were all significant on chlorophyll *b* (*p* < 0.05), while for the other responses, only factors A and B significantly influenced. The quadratic effects of starch (B^2^) and Arabic gum (C^2^) had no significant effect on the majority of responses except for weight loss. In contrast, the quadratic effect of *Aloe vera* was significant on almost all responses. The best interaction effects were those of *Aloe vera*-starch (AB) and *Aloe vera*-Arabic gum (AC), which were significant (*p* < 0.05) on almost all responses including chlorophyll *a*, chlorophyll *b*, carotenoids, RP, and TSS.

These regression coefficients were used to generate the prediction equations for the ripening parameters using the general formula of the *Y*_*i*_ polynomial equation. In connection to this, the coefficients of determination*R*^2^of chlorophyll *a* and chlorophyll *b* and carotenoids concentrations, firmness, TSS, PR, and WL responses were all higher than the standard value (75%). It provides an indication of whether there is a good fit between the prediction model and the experimental data. It has been suggested that a good model fit should have *R*^2^ close to 1 (*R*^2^ ≥ 0.75) [31]. These results indicated that there was good fit between the experimental data obtained and the responses predicted through the model. The model is therefore appropriate to explain the influence of *Aloe vera*, starch, and Arabic gum on the ripening parameters of banana.

The final prediction equations for chlorophyll *a* content, chlorophyll *b* content, carotenoid content, firmness, total soluble solid content, percentage of ripening, and weight loss of coated bananas as a function of the independent variables A, B, and C were as follows:

(5) $$\begin{array}{c} \text{Clorophyll }a\left(\text{mg}/\text{g}\right)=0.721-0.00807\text{ A}-0.00337\text{ B}+0.249\text{ C}+0.000024\;{\text{A}}^{2}+0.000006\;{\text{B}}^{2}+0.0193\;{\text{C}}^{2}+0.000019\text{ A}*\text{B}-0.001441\text{ A}*\text{C}-0.000636\text{ B}*\text{C},\\ \text{Chlorophyll b}\left(\text{mg}/\text{g}\right)=0.359-0.003466\text{ A}-0.001555\text{ B}+0.0748\text{ C}+0.000010\;{\text{A}}^{2}+0.000003\;{\text{B}}^{2}+0.0087\;{\text{C}}^{2}+0.000008\text{ A}*\text{B}-0.000483\text{ A}*\text{C}-0.000234\text{ B}*\text{C},\\ \text{Carotenoids }\left(\text{mg}/\text{g}\right)=0.574-0.00292\text{ A}-0.002985\text{ B}+0.0472\text{ C}+0.000003\;{\text{A}}^{2}+0.000004\;{\text{B}}^{2}-0.0315\;{\text{C}}^{2}+0.000015\text{ A}*\text{B}+0.000277\text{ A}*\text{C}-0.000157\text{ B}*\text{C},\\ \text{Firmness }\left(\text{N}\right)=4.92-0.0456\text{A}+0.0015\text{ B}+0.68\text{ C}+0.000126\;{\text{A}}^{2}+0.000010\;{\text{B}}^{2}-0.073\;{\text{C}}^{2}+0.000031\text{ A}*\text{B}-0.00020\text{ A}*\text{C}-0.00645\text{ B}*\text{C},\\ \text{Total soluble solids}\left({}^{\circ}\text{Brix}\right)=27.3+0.1715\text{ A}+0.0510\text{ B}-22.43\text{ C}-0.000810\;{\text{A}}^{2}+0.000017\;{\text{B}}^{2}+0.29\text{ C}2-0.000574\text{ A}*\text{B}+0.1002\text{ A}*\text{C}+0.0331\text{ B}*\text{C},\\ \text{Ripening Percentage }\left(\%\right)=20.9+1.277\text{ A}+0.229\text{ B}-63.4\text{ C}-0.003685\;{\text{A}}^{2}-0.000222\;{\text{B}}^{2}-0.18\;{\text{C}}^{2}-0.002985\text{ A}*\text{B}+0.2015\text{ A}*\text{C}+0.2349\text{ B}*\text{C}\\ \text{Weight loss}\left(\%\right)=11.46-0.0076\text{ A}-0.0179\text{ B}-3.97\text{ C}+0.000042\;{\text{A}}^{2}+0.000074\;{\text{B}}^{2}+1.523\;{\text{C}}^{2}-0.000057\text{ A}*\text{B}-0.00448\text{ A}*\text{C}+0.00529\text{ B}*\text{C}. \end{array}$$

### 3.3. Analysis of the Response Surfaces

#### 3.3.1. Pigments

**Figure 2:**
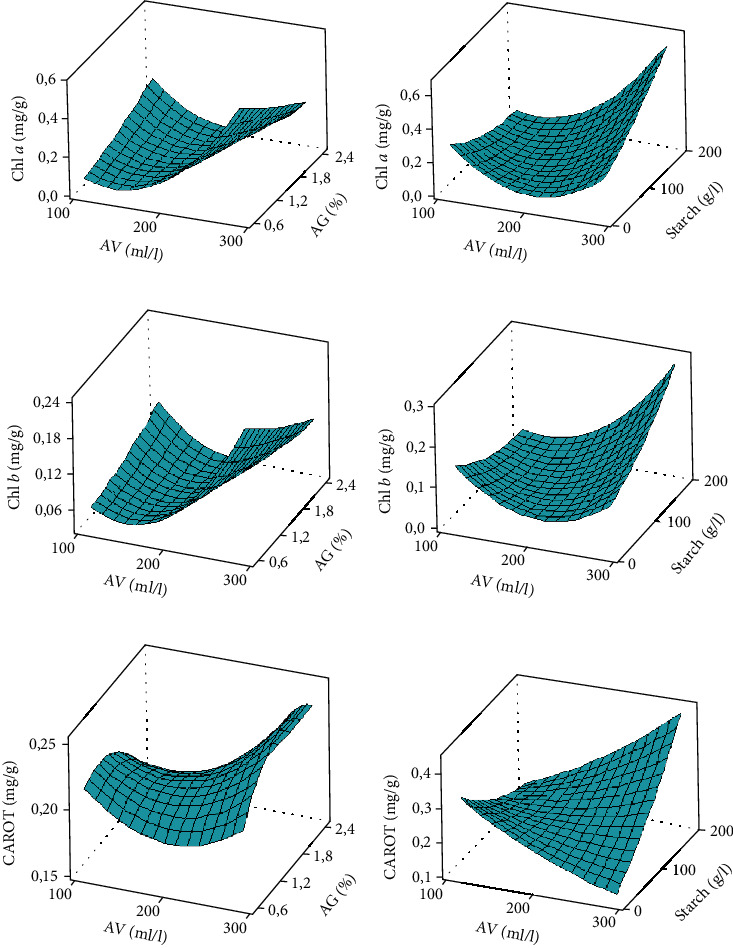
Chlorophyll *a*, chlorophyll *b*, and carotenoid response surface curves. AV: *Aloe vera*; AG: Arabic gum. Response surface curves made at 5% threshold statistical analysis.

Chlorophyll *a* and *b* contents were very high in bananas coated with high concentrations of *Aloe vera* (between 200 and 300 g/l) associated with low concentrations of Arabic gum (between 0.6 and 1.2%) (Figures 2(a)–2(d)). *Aloe vera*-Arabic gum as well as *Aloe vera*-starch interactions, more effectively maintained high concentration of photosynthetic pigment in the fruits. The same trend was observed with carotenoid content (Figures 2(e) and 2(f)). The lack of fit *p* value of <0.001 (Table 4) implies that it is significant compared to the pure error, but the coefficients of determination (*R*^2^) were greater than 91%, indicating a well-fitted response models and which shows that the combination of these three independent variables for the coating of bananas has a positive interaction on delaying pigment degradation. This result is similar to that of Rehman et al. [32] who showed that *Aloe vera* gel delayed chlorophyll degradation in guava during storage. Indeed, during the ripening process of fruits such as bananas, a change in color is observed as the consequence of chlorophyll degradation [33], and the appearance of carotenoids initially present in the chloroplasts as well as the release of precursors such as geraniol and mevalonic acid for the synthesis of new carotenoids [34]. The coated bananas experienced color change very late after the uncoated bananas. Starch and Arabic gum are known for their gelling power and film properties, to which could be attributed the delay in the degradation of chlorophyll and the reduction in gas exchange on the banana surface. Deng et al. [35] reported that the reduction and change in the internal gas composition of the fruit significantly delayed the breakdown of chlorophyll in bananas.

#### 3.3.2. Firmness

**Figure 3:**
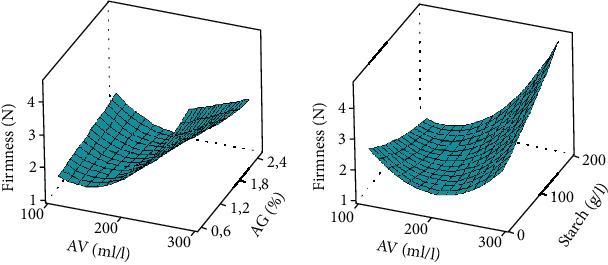
Firmness response surface curve. AV: *Aloe vera*; AG: Arabic gum. Response surface curve made at 5% threshold statistical analysis.

Several lots of coated bananas had higher firmness than uncoated bananas (Table 2).  Figure 3 shows that firmness is high at high concentrations of starch (between 100 and 200 g/l) and *Aloe vera* (between 200 and 300 g/l). Low concentrations of Arabic gum (0.6 and 1.8%) induced the high values of firmness. The interaction between *Aloe vera* and Arabic gum as well as between *Aloe vera* and starch more effectively maintained the firmness of coated bananas. The lack of fit was significant (*p* = 0.005), and the coefficient of determination (*R*^2^) was more than 84%, indicating a well-fitted response model. This shows that *Aloe vera*, starch, and Arabic gum delayed the loss of firmness of coated bananas. This result corroborates that of Donjio et al. [36] who observed the maintenance of the firmness of tomato fruits coated with a mixture of pineapple skin and Arabic gum, as well as that of Maqbool et al. [37] who showed that Arabic gum combined with 6% calcium chloride slowed down the loss of firmness in bananas during storage. The bioactive compounds present in the coating solutions may have affected the activities of hydrolase-type enzymes such as polygalacturonases, carboxymethylcellulases, and pectinmethylesterases by slowing down the degradation of cell wall components, enabling therefore to maintain high firmness in coated fruits. Firmness is a composite quality resulting from the combination of several factors such as turgidity and structural components of tissues and cells [27]. During ripening, the structural composition of the fruit is disturbed due to the hydrolysis of the molecules, which leads to the softening of the fruit [38, 39]. The hydrolysis process is enhanced by the osmotic migration of water from the epicarp to the pulp during banana ripening [40]. Pectins, celluloses, and hemicelluloses are the major classes of polysaccharides of the cell wall which contribute through their association to the firmness of the fruit. During ripening, these polysaccharides are solubilized, deesterified, and depolymerized, contributing to the loss of firmness of the fruit [41].

#### 3.3.3. Total Soluble Solid Contents (TSS)

**Figure 4:**
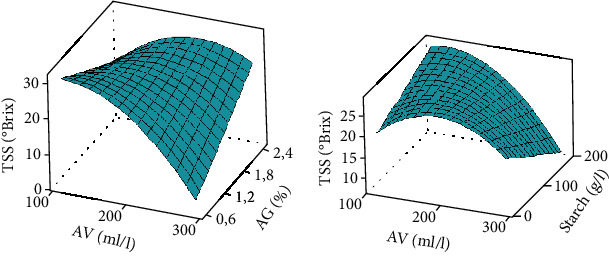
Total soluble solid response surface curve. AV: *Aloe vera*; AG: Arabic gum. Response surface curve made at 5% threshold statistical analysis.

Coated bananas showed the lowest total soluble solids values compared to uncoated bananas.  Figure 4 shows that the concentration ranges between 200 and 300 ml/l for *Aloe vera*, 0.6 and 1.2% for Arabic gum, and 100 and 200 g/l for starch are those that kept the TSS content of coated bananas low. The lack of fit was significant (*p* = 0.0001), and the coefficient of determination (*R*^2^) was more than 88%, indicating a well-fitted response model. The interactions between *Aloe vera*-Arabic gum and *Aloe vera*-starch are those that were the most effective in slowing down the process of TSS synthesis. Similar results were found by Kouete et al. [42] on mango fruits coated with a solution from cocoa leaf extract. Similarly, Mendy et al. [43] reported that papayas coated with *Aloe vera* extracts retain TSS better than uncoated fruits. Generally, the ripening of fruits such as bananas is associated by an increase in the TSS content [27]. In this study, the low levels of TSS could be due to the slowing down of the metabolic activities of the fruits caused by the control of gas exchanges under the effect of the coating. High values of TSS found in uncoated fruits could be due the hydrolysis of starch and other compounds into soluble sugar, acids, vitamin C, amino acids, and pectins [34, 44].

#### 3.3.4. Weight Loss (WL)

**Figure 5:**
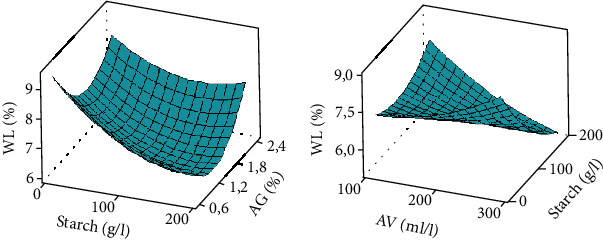
Weight loss surface curve. AV: *Aloe vera*; AG: Arabic gum. Response surface curve made at 5% threshold statistical analysis.

All coated bananas presented low percentages of physiological loss of mass as compared to the control. The lowest value of physiological loss of mass was obtained with 100 and 200 g/l starch and 0.6 and 1.2% Arabic gum (Figure 5). *Aloe vera* had no effect on the banana weight loss. The lack of fit was not significant (*p* = 0.292), and the coefficient of determination (*R*^2^) was more than 82%, indicating a well-fitted response model. The starch-Arabic gum interaction is the one that presented the lowest percentage of WL as compared to the *Aloe vera*-starch and *Aloe vera*-Arabic gum interactions. Previous study showed that weight loss can be reduced using cocoa leaf extracts on mangoes [27] or starch on banana [45]. Indeed, evapotranspiration and respiration are the processes that best explain the physiological loss of mass of fresh fruits during ripening [46]. The low physiological losses of mass of the treated bananas could be due to the reduction of transpiration in the coated fruits. Coating acts as a semipermeable barrier for O_2_, humidity, and for the movement of solutes. This results in the reduction of respiration, water loss, and oxidation reaction rate [47, 48].

#### 3.3.5. Ripening Percentage (RP)

**Figure 6:**
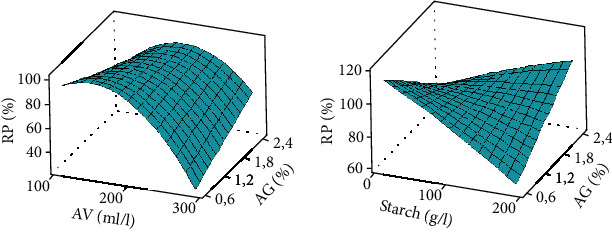
Ripening percentage surface curve. AV: *Aloe vera*; AG: Arabic gum. Response surface curve made at 5% threshold statistical analysis.

The coated bananas showed low ripening percentages, with a smaller value of 36.66% as compared to the control (100%) (Table 3). High concentrations of starch (100 to 200 g/l) and *Aloe vera* (200 to 300 ml/l) were found to be effective in maintaining low percentages of banana ripening rate (Figure 6). Likewise, the lower the Arabic gum concentration (0.6 to 1.2%), the lower the rate of ripening. The interactions between the *Aloe vera* and Arabic gum as well as that between starch and Arabic gum are those that had significant effects in reducing the ripening rate (Figure 6). The lack of fit was significant (*p* = 0.0001), and the coefficient of determination (*R*^2^) was more than 89%, indicating a well-fitted response model. Donjio et al. [36] found similar results to the low ripening rate of tomato coated with a solution made from Arabic gum and pineapple epicarp. The bioactive compounds such as the antioxidants in the coating solution in association with the enzymes may have slow down and/or inhibit the metabolic reactions involved in the ripening process such as the inhibition of chlorophyll degradation, TSS synthesis, loss of PPM, and firmness.

### 3.4. Validations of the Tests in Optimal Condition

The response values obtained under the optimal conditions predicted by the model are presented in Table 5. These results were confirmed by laboratory trials, and the optimal values were obtained. The statistical analysis showed that there is no significant difference between the predicted and experimental optimal values.

The optimum concentrations of *Aloe vera*, starch, and Arabic gum were reached at maximum values of chlorophyll *a*, chlorophyll *b*, and firmness and at minimum values of total soluble solids, percentage of ripening, weight loss, and carotenoid content. The results indicated that the calculated optimum concentrations of *Aloe vera*, starch, and Arabic gum are 286.799 ml.l^−1^, 102.589 g.l^−1^, and 1.0888% (*m*.*v*^−1^), respectively. An experimental verification done using the optimal concentrations obtained for the coating solution showed no significant difference between the experimental values and the calculated predicted values (Table 6).

## 4. Conclusion

In the aim of obtaining a composite coating allowing to extend the shelf life of bananas, the concentrations of *Aloe vera* gel, starch, and Arabic gum were optimized by the response surface methodology. Using a second-order polynomial equation for the fit, the responses (chlorophyll *a*, chlorophyll *b*, and carotenoids contents, firmness, weight loss, total soluble solid contents, and percentage of ripening) embodying ripening were predicted. Under optimal conditions, chlorophyll *a*, chlorophyll *b*, and firmness were maximal, while weight loss, total soluble solids, and percentage of ripening were minimal. Therefore, the interactions between *Aloe vera*-starch and *Aloe vera*-Arabic gum are those that have most influenced the responses. The optimal concentrations of the different factors for good fruit preservation were 286.799 ml.l^−1^*Aloe vera*, 102.589 g.l^−1^ starch, and 1.0888% (*m*.*v*^−1^) Arabic gum. However, the formulation of a coating from the combination of these three concentrations is proposed as a simple and effective method for better postharvest preservation of bananas.

## Figures and Tables

**Table 1:** Independent variables.

Variables	Levels				
	Code	(−*α*) = −1.809	Minimum	Maximum	(+*α*) = +1.809
*Aloe vera* (ml)	A	109.547	150	250	290.453
Starch (g)	B	9.546	50	150	190.453
Arabic gum (%)	C	0.595	1	2	2.404

**Table 2:** Experimental design.

Treatment *N*°	*Aloe vera* (ml)	Starch (g)	AG (%)
Control	0	0	0
T1	109.546	100	1.5
T2c	200	100	1.5
T3	200	190.453	1.5
T4c	200	100	1.5
T5	200	100	2.404
T6	250	150	2
T7	150	50	1
T8c	200	100	1.5
T9	250	50	2
T10c	200	100	1.5
T11c	200	100	1.5
T12	250	150	1
T13	150	150	2
T14	290.453	100	1.5
T15	250	50	1
T16	200	100	0.595
T17	150	50	2
T18	150	150	1
T19	200	9.5465	1.5
T20c	200	100	1.5

c: center point. AG: Arabic gum.

**Table 3:** Results obtained following the experimental design.

Tests	*Aloe vera* (ml)	Starch (g)	GA (%)	CHLA (mg/g)	CHLB (mg/g)	CAROT (mg/g)	Firmness (N)	TSS (°Brix)	RP (%)	Weight loss (%)
Control	*/*	/	/	0.047 ± 0.003^a^	0.031 ± 0.008^r^	0.286 ± 0.002^s^	1.370 ± 0.083^b^	25.910 ± 0.305^r^	100 ± 0.00^j^	10.450 ± 0.401^o^
1	109.546	100	1.5	0.123 ± 0.01^n^	0.068 ± 0.006^f^	0.229 ± 0.001^n^	1.437 ± 0.182^e^	25.800 ± 0.529^p^	83.333 ± 1.033^e^	7.188 ± 0.155^i^
2c	200	100	1.5	0.072 ± 0.001^c^	0.049 ± 0.005^m^	0.206 ± 0.001^j^	1.573 ± 0.201^f^	25.067 ± 0.251^k^	90.000 ± 0.00^g^	6.950 ± 0.602^g^
3	200	190.453	1.5	0.144 ± 0.011^o^	0.081 ± 0.007^d^	0.300 ± 0.004^t^	1.763 ± 0.180^n^	25.733 ± 0.338^n^	90.000 ± 0.00^g^	6.469 ± 0.226^e^
4c	200	100	1.5	0.072 ± 0.001^c^	0.040 ± 0.008^q^	0.200 ± 0.001^g^	1.400 ± 0.135^c^	25.100 ± 0.441^l^	90.000 ± 0.00^g^	6.510 ± 0.111^e^
5	200	100	2.404	0.087 ± 0.004^i^	0.051 ± 0.009^k^	0.200 ± 0.006^g^	1.427 ± 0.328^d^	25.867 ± 0.033^q^	86.667 ± 1.342^f^	7.555 ± 0.334^lm^
6	250	150	2	0.191 ± 0.016^q^	0.094 ± 0.004^c^	0.246 ± 0.007^p^	1.640 ± 0.241^h^	24.433 ± 0.185^f^	75.430 ± 2.010^c^	6.911 ± 0.592^g^
7	150	50	1	0.106 ± 0.019^m^	0.056 ± 0.005^j^	0.231 ± 0.001^o^	1.683 ± 0.094^j^	25.767 ± 0.288^o^	91.540 ± 1.673^h^	7.036 ± 0.122^h^
8c	200	100	1.5	0.076 ± 0.007^e^	0.044 ± 0.007^n^	0.209 ± 0.002^m^	1.700 ± 0.225^l^	25.100 ± 0.318^l^	90.000 ± 0.00^g^	5.921 ± 0.255^a^
9	250	50	2	0.086 ± 0.005^h^	0.042 ± 0.006^p^	0.168 ± 0.001^c^	1.840 ± 0.132^r^	25.567 ± 0.145^m^	83.333 ± 1.902^e^	7.402 ± 0.155^k^
10c	200	100	1.5	0.068 ± 0.004^b^	0.043 ± 0.002^o^	0.207 ± 0.003^k^	1.620 ± 0.271^g^	25.000 ± 0.333^i^	90.000 ± 0.00^g^	6.170 ± 0.572^c^
11c	200	100	1.5	0.072 ± 0.003^c^	0.043 ± 0.009^o^	0.208 ± 0.005^l^	1.660 ± 0.013^i^	25.050 ± 0.166^j^	90.000 ± 0.00^g^	6.308 ± 0.144^d^
12	250	150	1	0.427 ± 0.014^s^	0.172 ± 0.125^b^	0.279 ± 0.004^r^	2.262 ± 0.300^s^	12.500 ± 0.206^b^	43.333 ± 2.708^b^	6.128 ± 0.601^bc^
13	150	150	2	0.098 ± 0.007^l^	0.044 ± 0.007^n^	0.160 ± 0.003^b^	1.339 ± 0.124^a^	23.900 ± 0.09^d^	86.667 ± 1.660^f^	7.116 ± 0.333^i^
14	290.453	100	1.5	0.420 ± 0.012^r^	0.189 ± 0.004^a^	0.265 ± 0.006^q^	4.000 ± 0.00^t^	11.000 ± 0.201^a^	36.667 ± 1.970^a^	6.073 ± 0.232^b^
15	250	50	1	0.153 ± 0.015^p^	0.076 ± 0.003^e^	0.142 ± 0.002^a^	1.690 ± 0.156^k^	22.020 ± 0.187^c^	90.000 ± 0.00^g^	7.851 ± 0.186^m^
16	200	100	0.595	0.089 ± 0.002^j^	0.058 ± 0.006^h^	0.192 ± 0.001^f^	1.833 ± 0.009^q^	24.667 ± 0.123^h^	93.333 ± 1.091^i^	7.504 ± 0.276^l^
17	150	50	2	0.077 ± 0.001^f^	0.050 ± 0.007^l^	0.186 ± 0.001^e^	1.727 ± 0.165^m^	24.367 ± 0.190^e^	80.000 ± 0.00^h^	7.738 ± 0.188^n^
18	150	150	1	0.085 ± 0.007^g^	0.052 ± 0.008^j^	0.177 ± 0.003^d^	1.813 ± 0.230^p^	27.060 ± 0.202^s^	90.000 ± 0.00^g^	6.588 ± 0.192^f^
19	200	9.5465	1.5	0.096 ± 0.006^k^	0.060 ± 0.009^g^	0.201 ± 0.004^h^	1.780 ± 0.133^o^	24.600 ± 0.300^g^	86.667 ± 0.801^f^	7.310 ± 0.400^j^
20c	200	100	1.5	0.074 ± 0.004^d^	0.042 ± 0.004^p^	0.205 ± 0.001^i^	1.400 ± 0.283^c^	25.100 ± 0.231^l^	90.000 ± 0.00^g^	6.265 ± 0.277^d^
*F* value				306.63	38.90	15.52	18.39	179.52	59501.57	374.99
*p* value				<0.001	<0.001	<0.001	<0.001	<0.001	<0.001	<0.001
D.f.				62	62	62	62	62	62	62

c = center point. Values follow by the same letter(s) in each column are not statistically different at a threshold of 5%. CHLA: chlorophyll *a*; CHLB: chlorophyll *b*; CAROT: carotenoids; TSS: total soluble solids; RP: ripening percentage; D.f.: degree of freedom.

**Table 4:** Regression coefficients, coefficients of determination (*R*^2^), and *p* value of lack of fit of predicted equations.

Responses	Source	CR	*p* values	Lack of fit (*p* value)	*R* ^2^
Chlorophyll *a* (mg/g)	A	-0.00807	<0.0001^∗^	<0.0001^∗^	91.96%
	B	-0.00337	0.014^∗^		
	C	0.249	0.066		
	A^2^	0.000024	<0.0001^∗^		
	B^2^	0.000006	0.165		
	C^2^	0.0193	0.628		
	AB	0.000019	0.008^∗^		
	AC	-0.001441	0.033^∗^		
	BC	-0.000636	0.3		
	Constant	0.721	—		

Chlorophyll *b* (mg/g)	A	-0.003466	<0.0001^∗^	<0.0001^∗^	93.70%
	B	-0.001555	0.009^∗^		
	C	0.0748	0.028^∗^		
	A^2^	0.00001	<0.0001^∗^		
	B^2^	0.000003	0.064		
	C^2^	0.0087	0.532		
	AB	0.000008	0.003^∗^		
	AC	-0.000483	0.038^∗^		
	BC	-0.000234	0.272		
	Constant	0.359	—		

Carotenoids (mg/g)	A	-0.00292	0.129	<0.0001^∗^	81.73%
	B	-0.002985	0.005^∗^		
	C	0.0472	0.547		
	A^2^	0.000003	0.188		
	B^2^	0.000004	0.136		
	C^2^	-0.0315	0.179		
	AB	0.000015	0.001^∗^		
	AC	0.000277	0.416		
	BC	-0.000157	0.64		
	Constant	0.574			

Firmness (N)	A	-0.0447	0.005^∗^	0.005^∗^	85.36%
	B	-0.0021	0.957		
	C	2.16	0.312		
	A^2^	0.000136	0.008^∗^		
	B^2^	0.00002	0.797		
	C^2^	0.032	0.852		
	AB	0.000098	0.593		
	AC	-0.00677	0.972		
	BC	-0.01313	0.284		
	Constant	4.92			

Total soluble solids (°Brix)	A	0.1715	<0.0001^∗^	<0.0001^∗^	88.10%
	B	0.051	0.332		
	C	-22.43	0.117		
	A^2^	-0.000810	0.002^∗^		
	B^2^	0.000017	0.93		
	C^2^	0.29	0.88		
	AB	-0.000574	0.07		
	AC	0.1002	0.005^∗^		
	BC	0.0331	0.268		
	Constant	27.3	—		

Ripening percentage (%)	A	1.277	<0.0001^∗^	<0.0001^∗^	89.67%
	B	0.229	0.124		
	C	-63.4	0.955		
	A^2^	-0.003685	<0.0001^∗^		
	B^2^	-0.000222	0.735		
	C^2^	-0.18	0.978		
	AB	-0.002985	0.011^∗^		
	AC	0.2015	0.062		
	BC	0.2348	0.034^∗^		
	Constant	20.9	—		

Weight loss (%)	A	0.0114	0.16	0.292	82.48%
	B	-0.0041	0.008^∗^		
	C	-2.61	0.281		
	A^2^	0.000042	0.264		
	B^2^	0.000073	0.066		
	C^2^	1.516	0.002^∗^		
	AB	-0.000132	0.314		
	AC	-0.01200	0.425		
	BC	0.00482	0.349		
	Constant	11.46	—		

∗Significant at 0.05 level. CR: regression coefficients; *R*^2^: coefficients of determination.

**Table 5:** Predicted and experimental values of individual optimization of each response.

Response	*Aloe vera*	Starch	AG	Optimal predicted values	Optimal experimental values	Desirability
CHLA (mg/g)	290.4535	190.4535	0.595465	0.8862	0.8931 ± 0.01	1.00
CHLB (mg/g)	290.4535	190.4535	0.595465	0.3663	0.3681 ± 0.02	1.00
CAROT (mg/g)	290.4535	9.5465	0.5954	0.0622	0.0580 ± 0.006	1.00
Firmness (N)	290.4535	190.4535	0.5954	4	3.9133 ± 0.1	1.00
TSS (°Brix)	253.5396	190.4535	0.595465	7.0	7.266 ± 0.568	1.00
PR (%)	250.8760	172.8464	0.8390	36	35.733 ± 0.305	1.00
WL (%)	290.4535	190.4535	1.7101	5.0826	5.143 ± 0.14	1.00

AG: Arabic gum. Analysis of variance was made at a threshold of 5%.

**Table 6:** Predicted and experimental values of multiple optimization.

Responses	Optimal predicted values	Optimal experimental values	*p* value	Desirability
CHLA (mg/g)	0.4582	0.4460 ± 0.015	0.251	
CHLB (mg/g)	0.1943	0.1907 ± 0.001	0.071	
CAROT (mg/g)	0.2372	0.2347 ± 0.021	0.849	0.8651
Firmness (N)	3.8535	3.9333 ± 0.115	0.297	
TSS (°Brix)	9.3389	10.13 ± 1.00	0.241	
RP (%)	37.2362	36.63 ± 0.41	0.065	
WL (%)	7.0191	7.43 ± 0.40	0.157	

Analysis of variance was made at a threshold of 5%.

## Data Availability

The data shall be made available upon request to the corresponding author.
